# Supplementary material for: Depleting chemoresponsive mitochondrial fission mediator DRP1 does not mitigate sarcoma resistance
Source: Life Sci Alliance. 2024 Dec 6;8(2):e202402870. doi: 10.26508/lsa.202402870 (PMC11629689; doi:10.26508/lsa.202402870)

Figure 1C

Uncropped images of experiments displayed in Fig. 1C are followed by other biological replicates. Sample order same as displayed in the figure if not indicated differently.

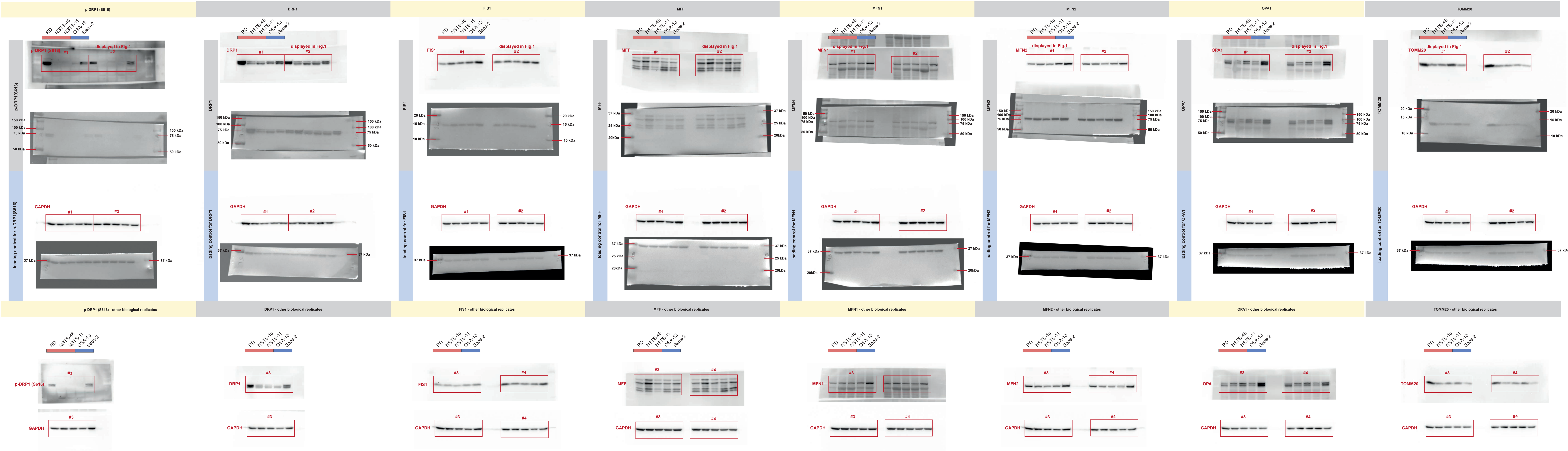

Supplement: Supplementary file 1 [file LSA-2024-02870_SdataF1.pdf]
