# Supplementary material for: Depleting chemoresponsive mitochondrial fission mediator DRP1 does not mitigate sarcoma resistance
Source: Life Sci Alliance. 2024 Dec 6;8(2):e202402870. doi: 10.26508/lsa.202402870 (PMC11629689; doi:10.26508/lsa.202402870)

Figure 4A, part I.

Uncropped images of experiments displayed in Fig. 4A are followed by other biological replicates. Sample order same as displayed in the figure if not indicated differently.

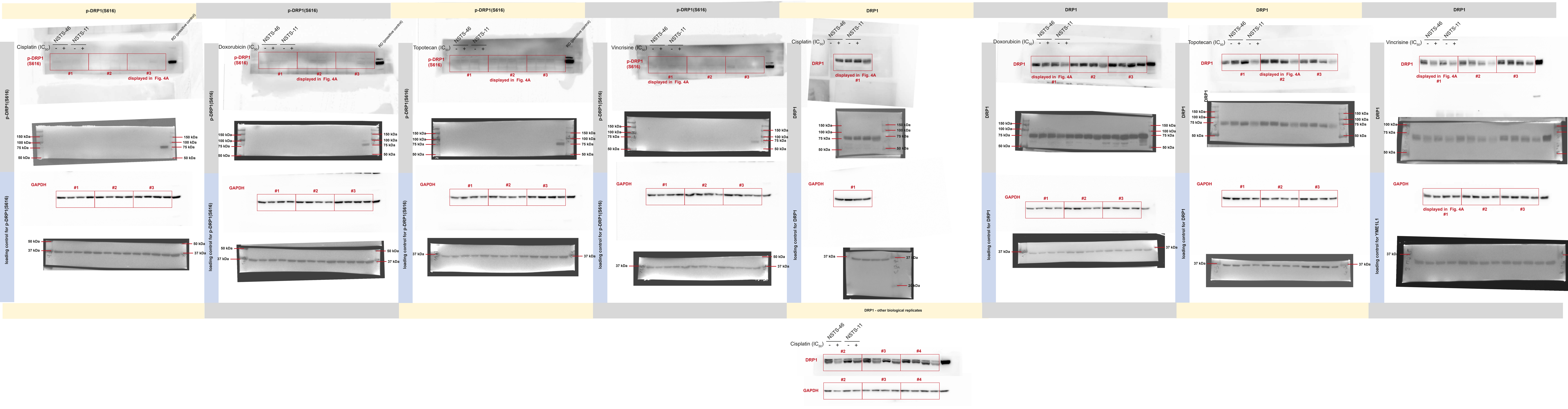

Figure 4A, part II.

Uncropped images of experiments displayed in Fig. 4A are followed by other biological replicates. Sample order same as displayed in the figure if not indicated differently.

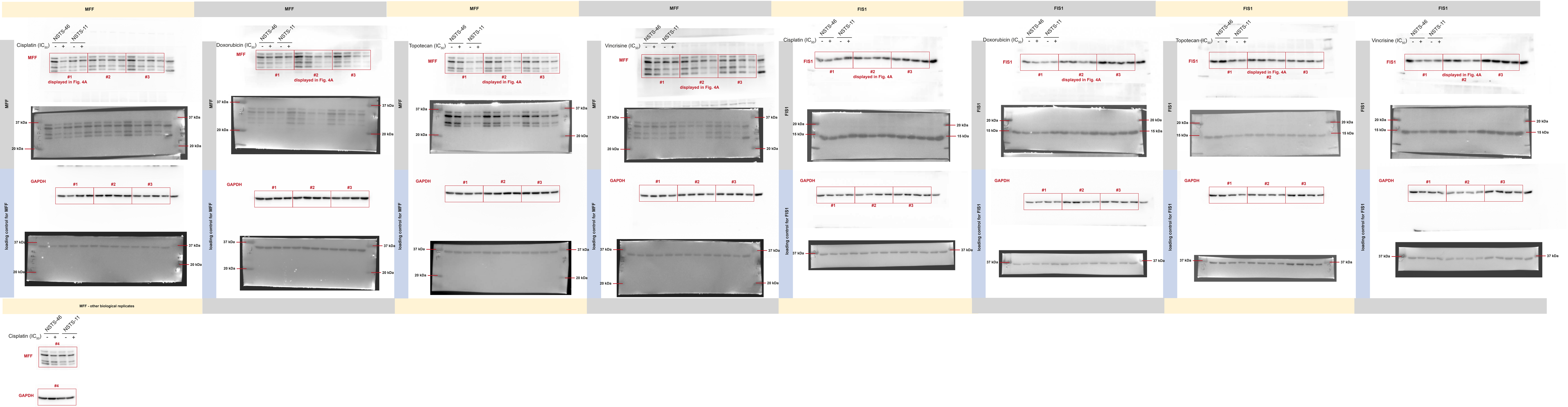

Figure 4A, part III.

Uncropped images of experiments displayed in Fig. 4A are followed by other biological replicates. Sample order same as displayed in the figure if not indicated differently.

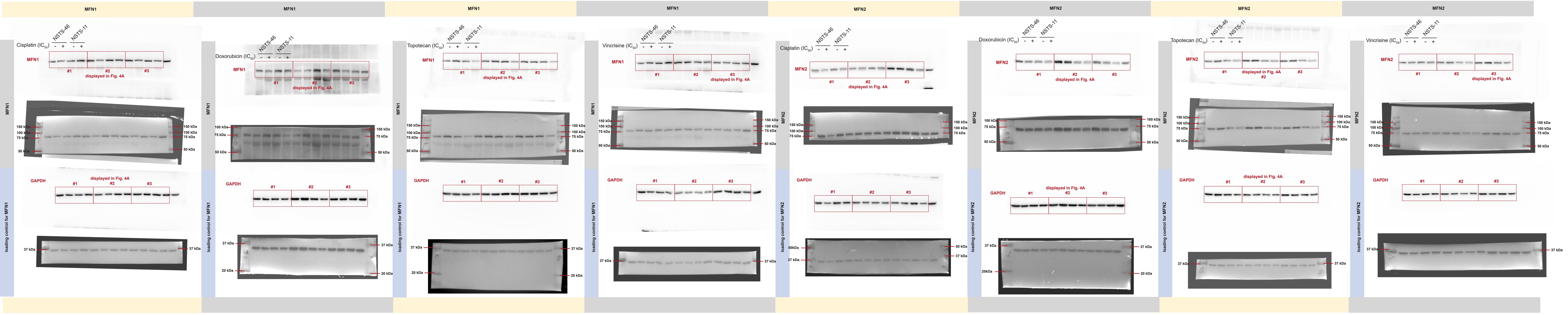

Figure 4A, part IV.

Uncropped images of experiments displayed in Fig. 4A are followed by other biological replicates. Sample order same as displayed in the figure if not indicated differently.

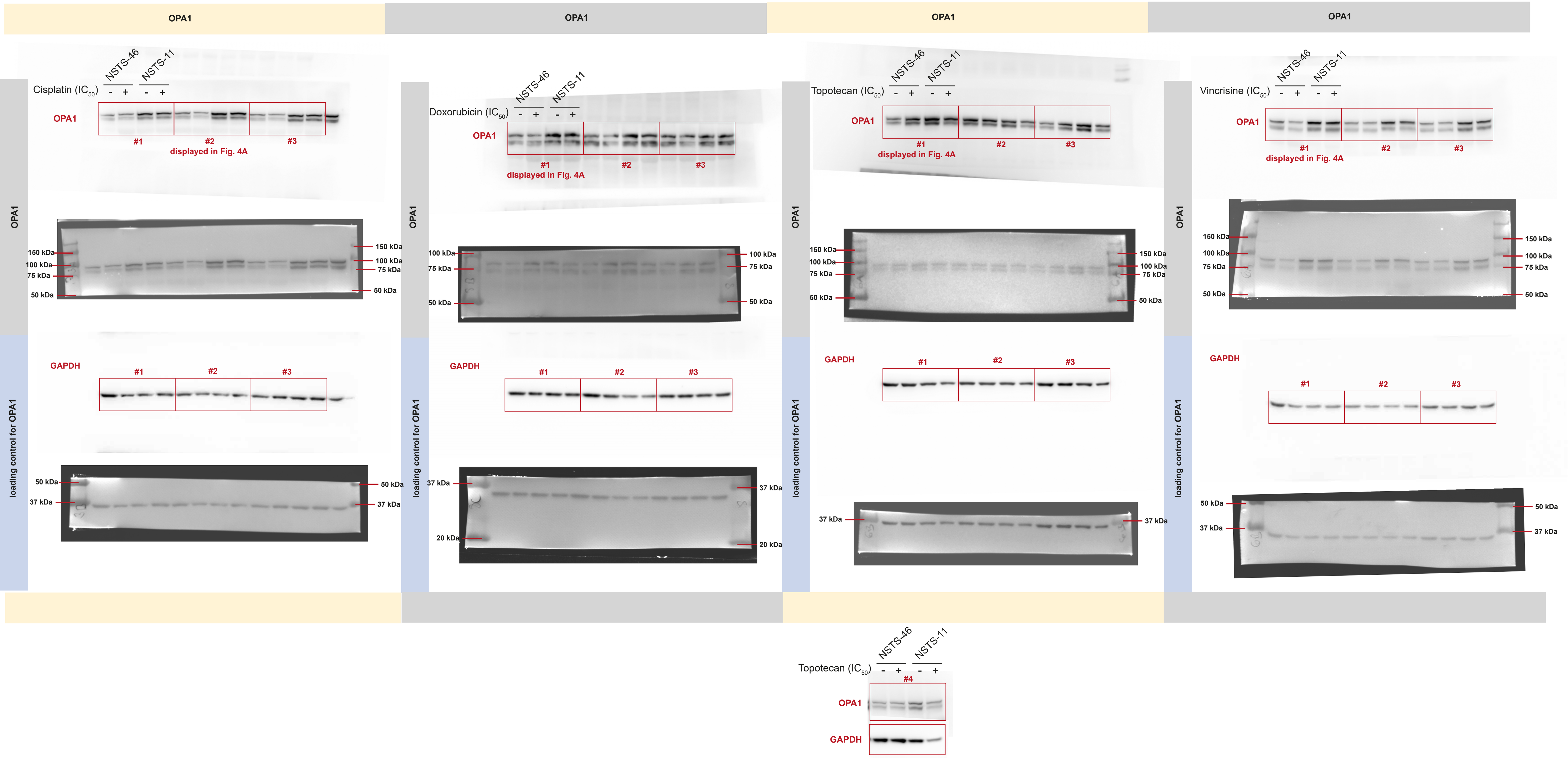

Figure 4B, part I.

Uncropped images of experiments displayed in Fig. 4B are followed by other biological replicates. Sample order same as displayed in the figure if not indicated differently.

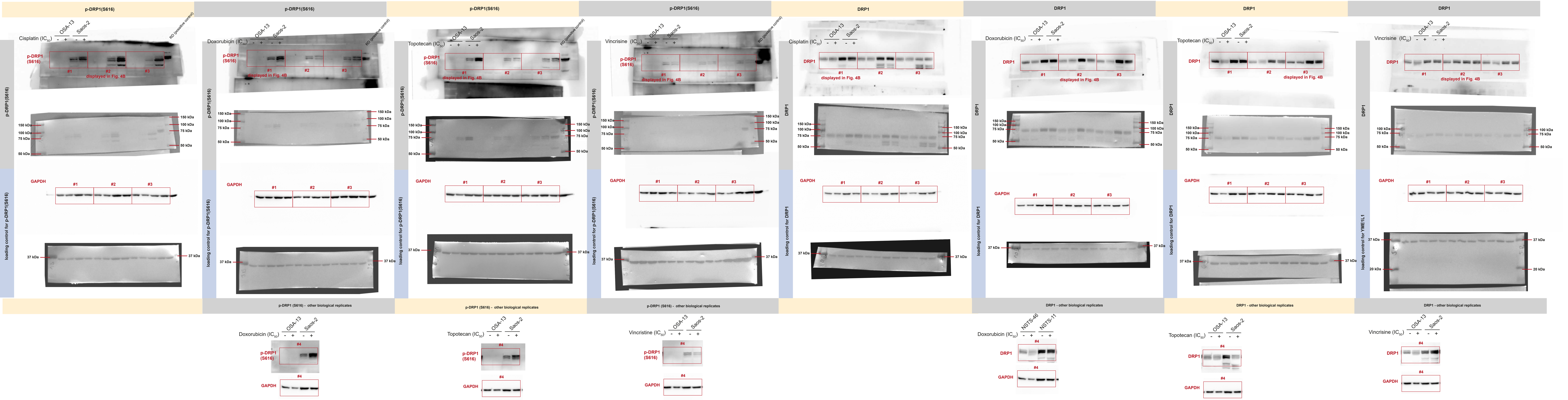

Figure 4B, part II.

Uncropped images of experiments displayed in Fig. 4B are followed by other biological replicates. Sample order same as displayed in the figure if not indicated differently.

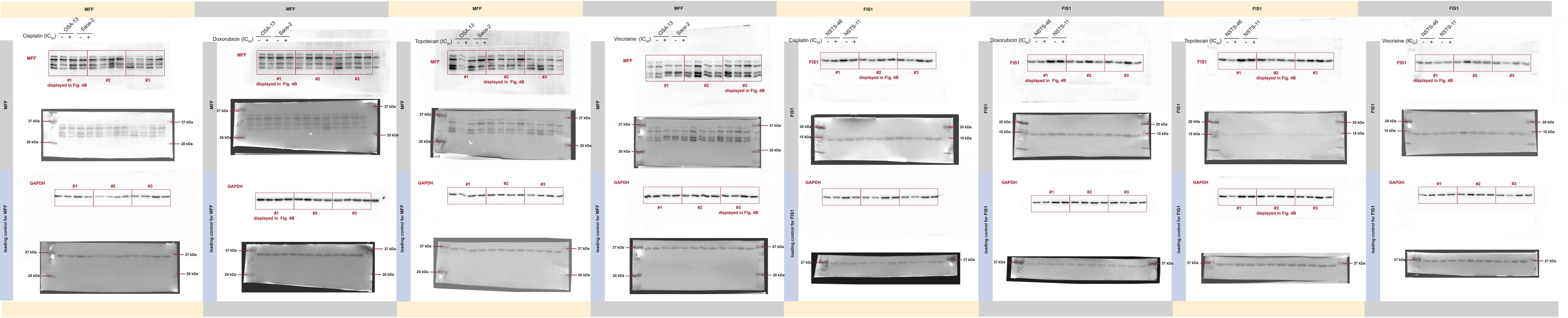

Figure 4B - right, part III.

Uncropped images of experiments displayed in Fig. 4B are followed by other biological replicates. Sample order same as displayed in the figure if not indicated differently.

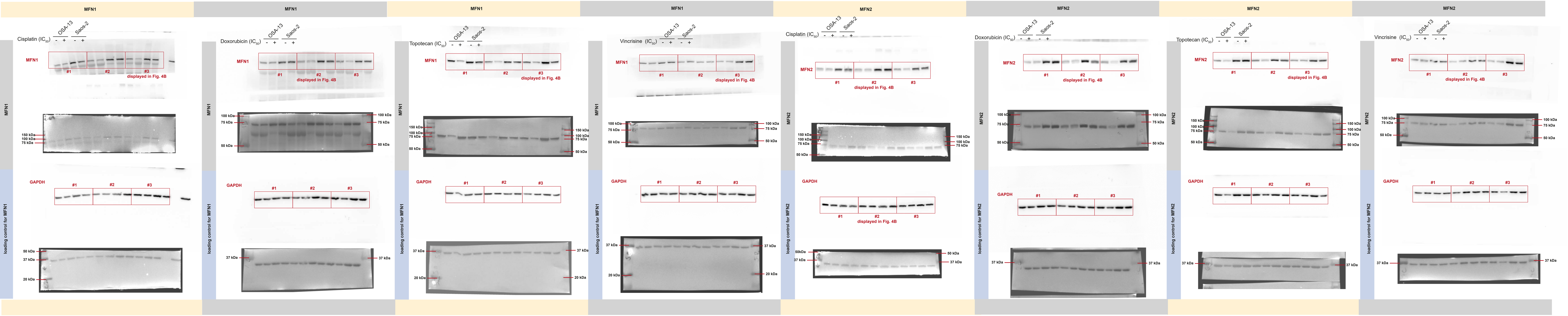

Figure 4B, part IV.

Uncropped images of experiments displayed in Fig. 4B are followed by other biological replicates. Sample order same as displayed in the figure if not indicated differently.

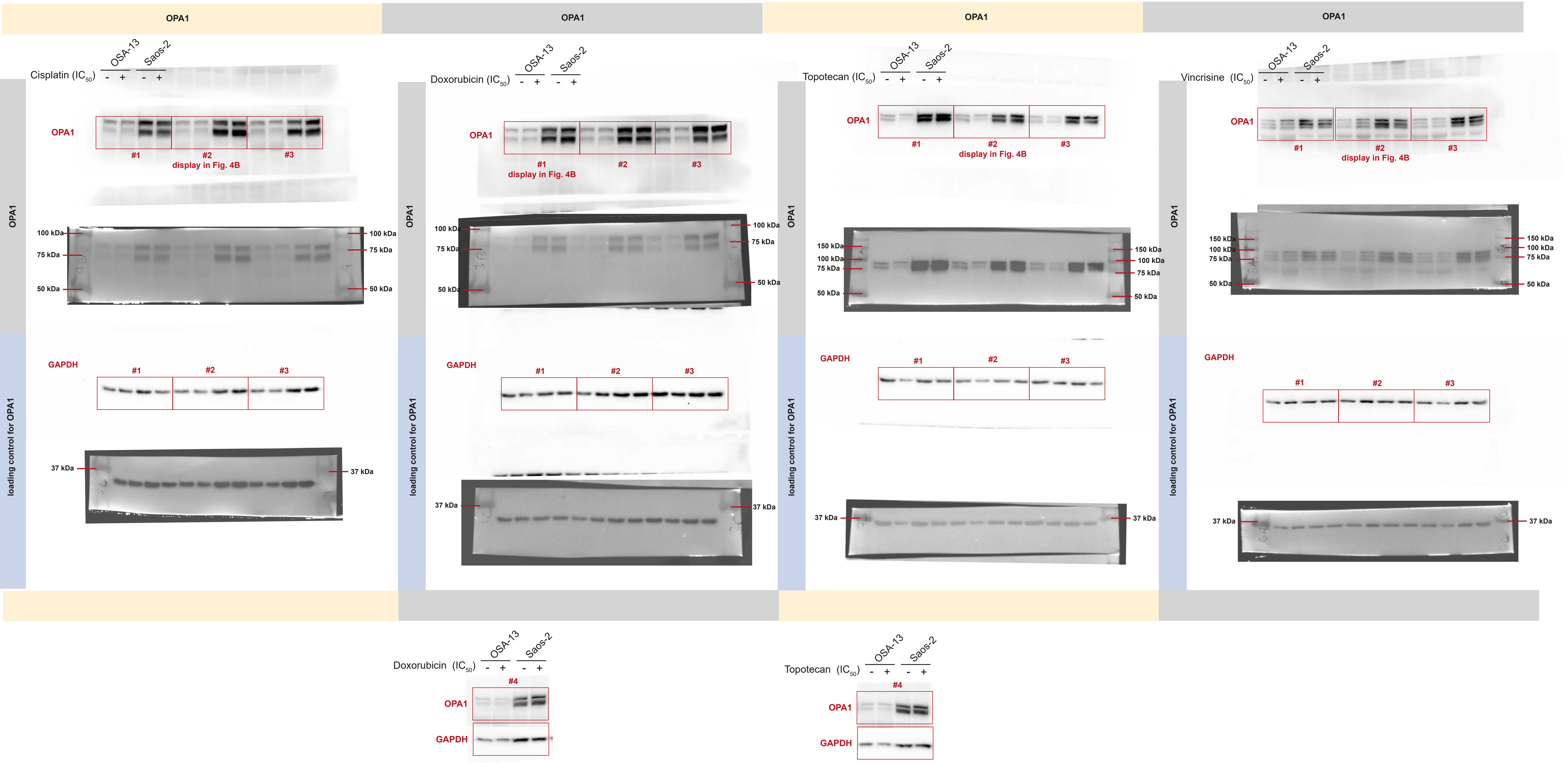

Supplement: Supplementary file 3 [file LSA-2024-02870_SdataF4_FS5.pdf]
