# Supplementary material for: Depleting chemoresponsive mitochondrial fission mediator DRP1 does not mitigate sarcoma resistance
Source: Life Sci Alliance. 2024 Dec 6;8(2):e202402870. doi: 10.26508/lsa.202402870 (PMC11629689; doi:10.26508/lsa.202402870)

Figure S6A - left

Uncropped images of experiments displayed in Fig. S6A are followed by other biological replicates. Sample order same as displayed in the figure if not indicated differently.

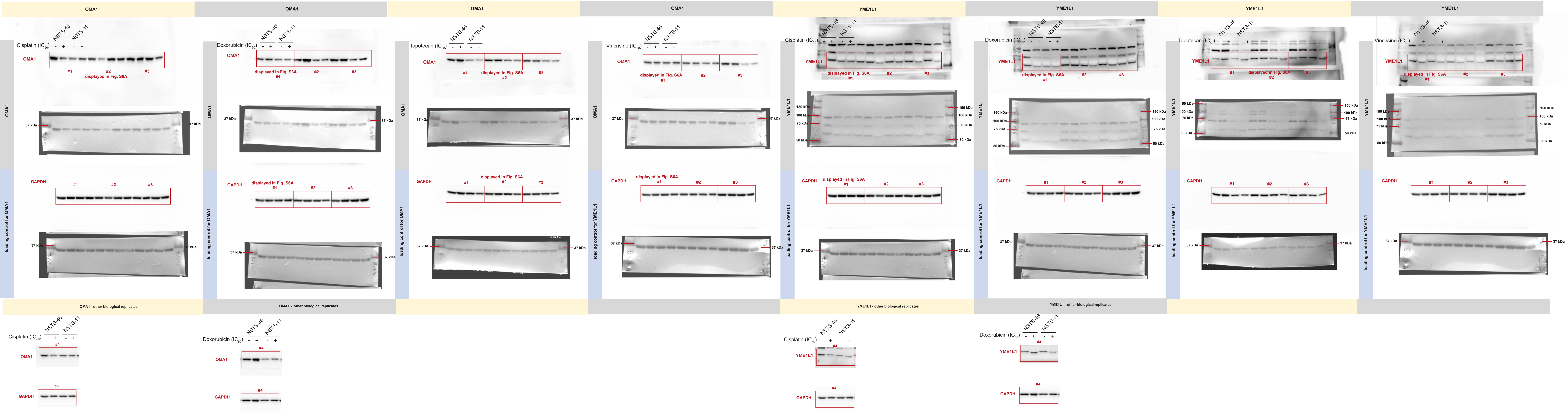

Figure S6A - right

Uncropped images of experiments displayed in Fig. S6A are followed by other biological replicates. Sample order same as displayed in the figure if not indicated differently.

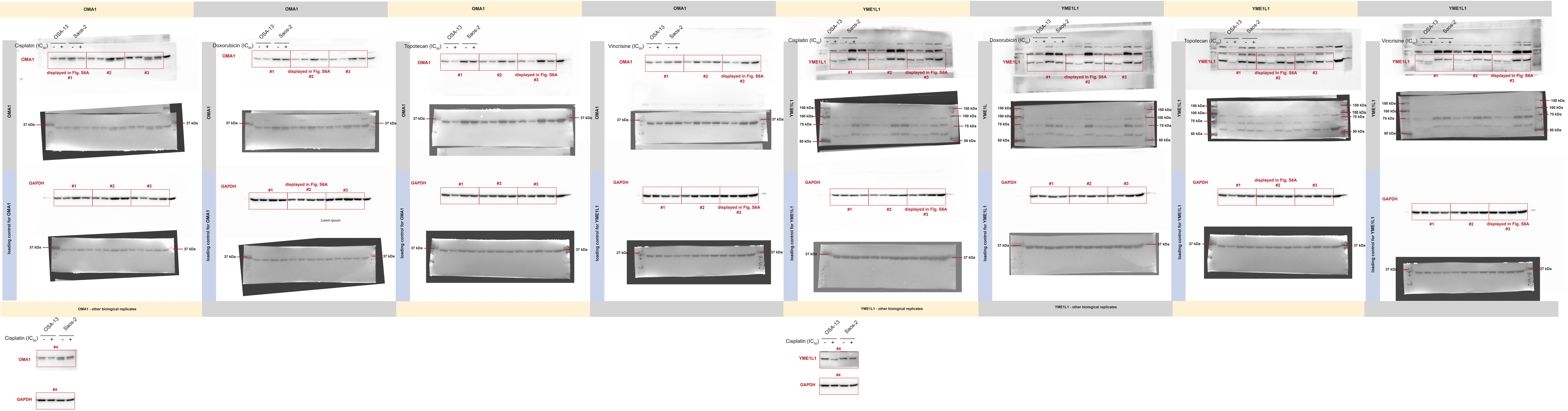

Figure S6B

Uncropped images of experiments displayed in Fig. S6B are followed by other biological replicates. Sample order same as displayed in the figure if not indicated differently.

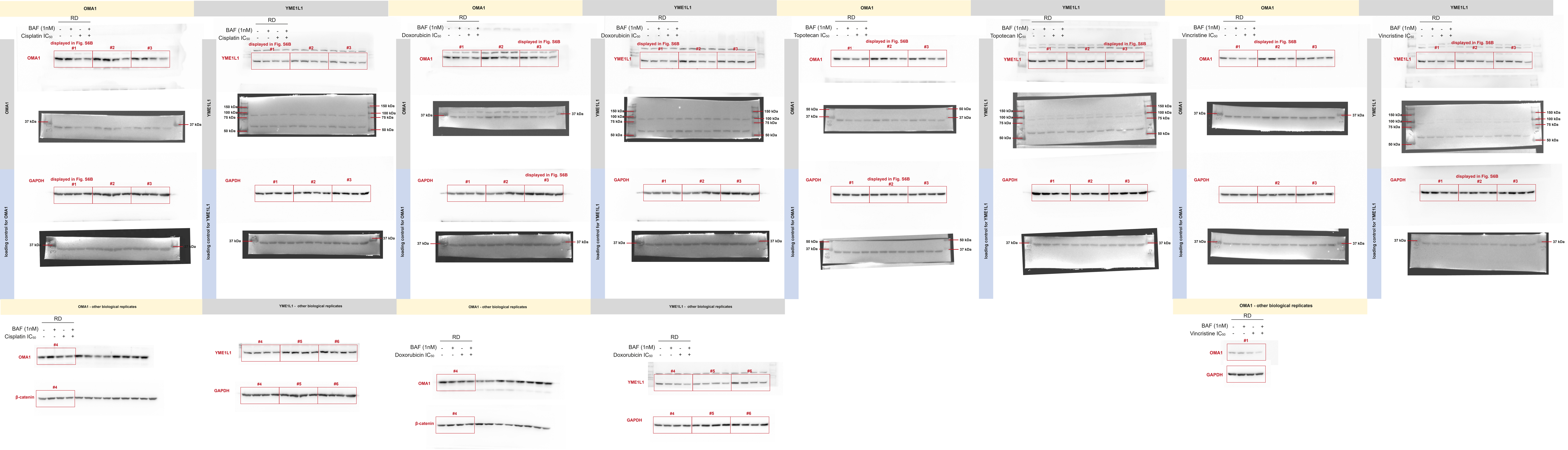

Supplement: Supplementary file 4 [file LSA-2024-02870_SdataFS6.pdf]
