# Supplementary material for: Depleting chemoresponsive mitochondrial fission mediator DRP1 does not mitigate sarcoma resistance
Source: Life Sci Alliance. 2024 Dec 6;8(2):e202402870. doi: 10.26508/lsa.202402870 (PMC11629689; doi:10.26508/lsa.202402870)

Figure 8A

Uncropped images of experiments displayed in Fig. 8A - are followed by other biological replicates. Sample order same as displayed in the figure if not indicated differently.

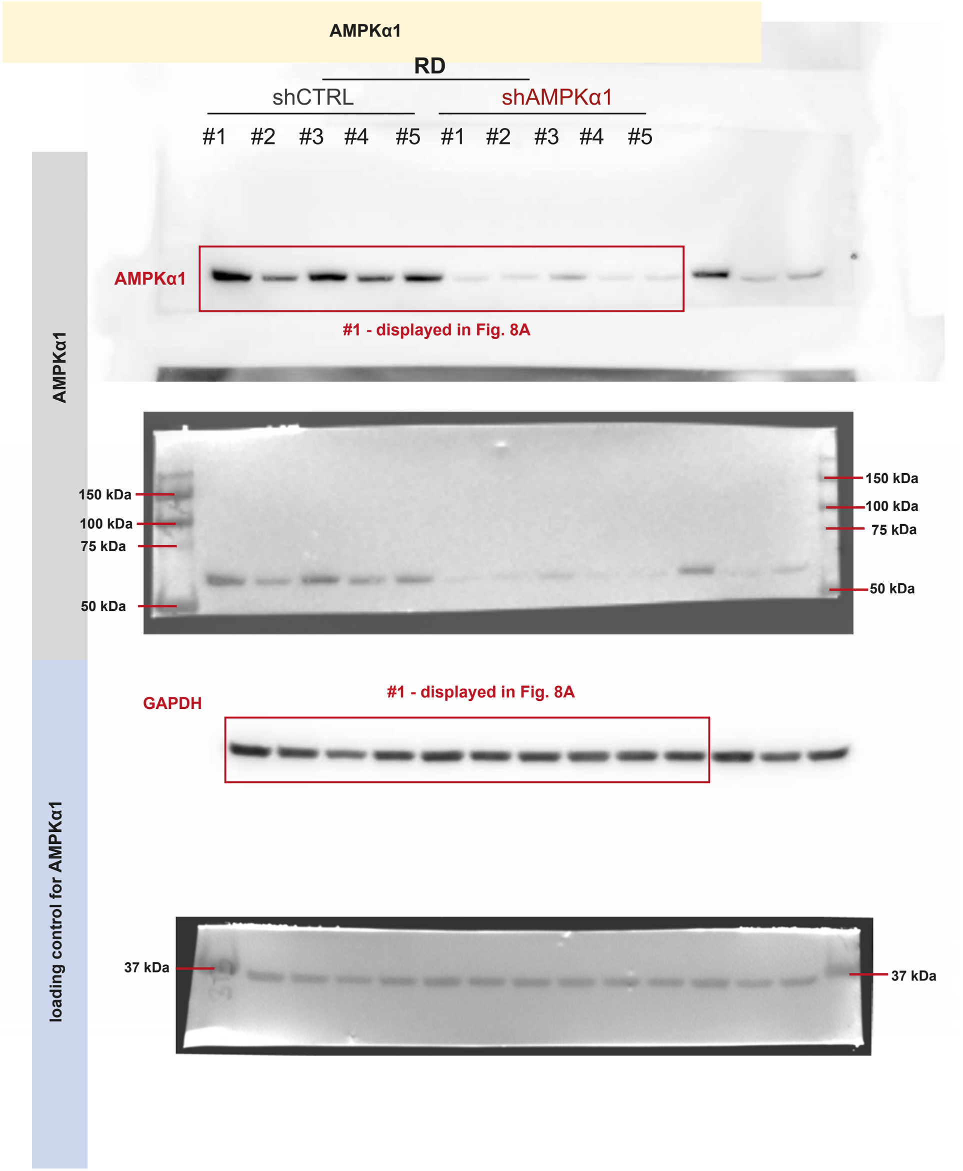

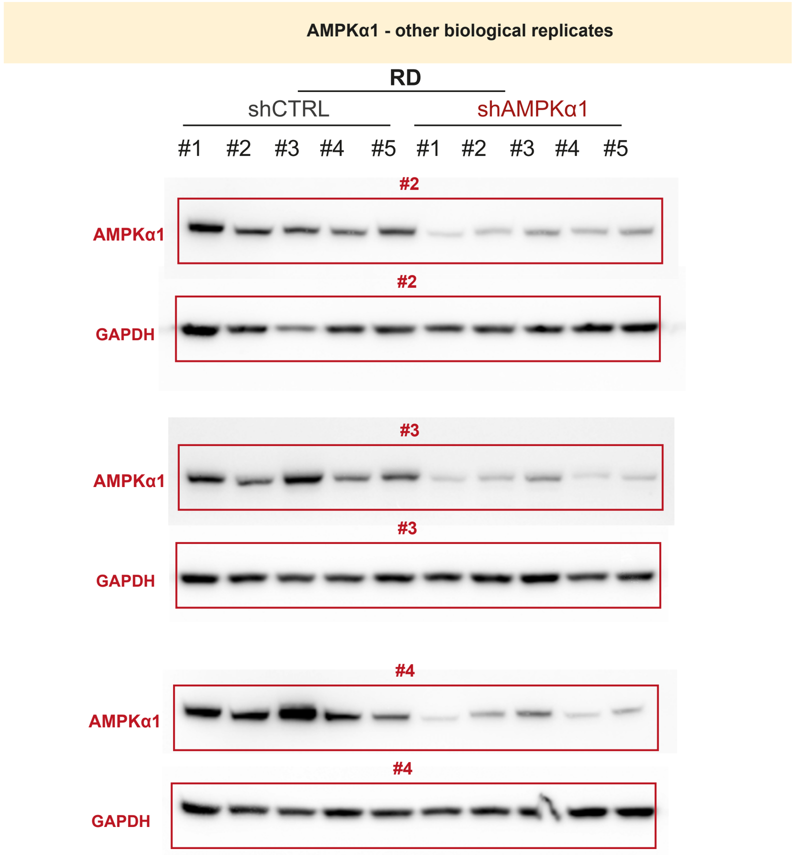

Figure 8B

Uncropped images of experiments displayed in Fig. 8B are followed by other biological replicates. Sample order same as displayed in the figure if not indicated differently.

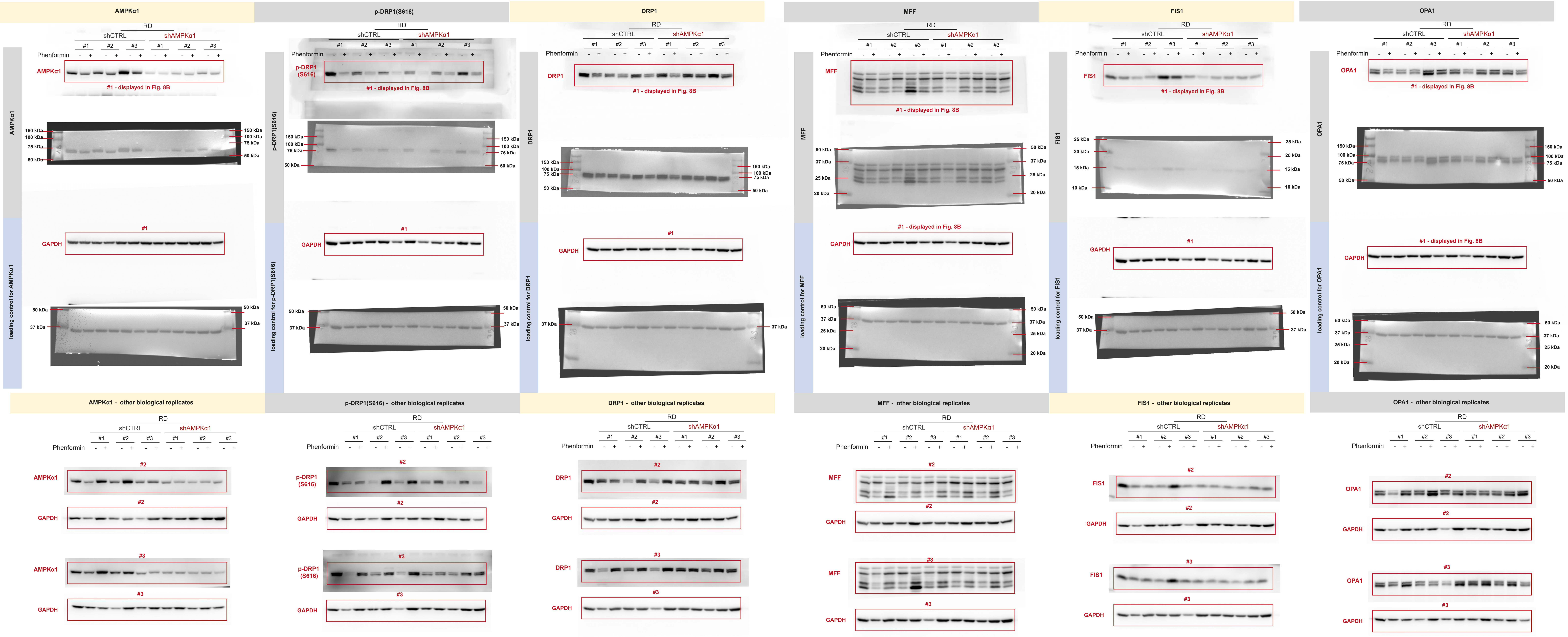

Supplement: Supplementary file 8 [file LSA-2024-02870_SdataF8_FS19.pdf]
