# Supplementary material for: Depleting chemoresponsive mitochondrial fission mediator DRP1 does not mitigate sarcoma resistance
Source: Life Sci Alliance. 2024 Dec 6;8(2):e202402870. doi: 10.26508/lsa.202402870 (PMC11629689; doi:10.26508/lsa.202402870)
Supplement: Supplementary file 14 [file LSA-2024-02870_TableS1.docx]

**Supplementary Table 1. Summary of cell lines and culture media composition**

| **Cell type** | **Cell Line** | **Cellosaurus Accession #** | | **Source** | **Therapy**  **Status*** | **Culture**  **Medium** | **Supplements** |
| --- | --- | --- | --- | --- | --- | --- | --- |
| Osteosarcoma | Saos-2 | CVCL_0548 | | ECACC | Post | DMEM Low Glucose (LM‑D1100, Biosera) | 10% FBS (FB-1101); 2 mM L‑Glutamine (XC-T1715); Penicillin (100 IU/ml) /Streptomycin (100 μg/ml) (XC‑A4122) (all Biosera) |
|  | OSA-13 | In-house derived (under IGA MZCR NR/9125-4 project; ethics approval no. 23/2005) | | | Pre |  | 20% FBS (FB-1101); 2 mM L‑Glutamine (XC-T1715); Penicillin (100 IU/ml) /Streptomycin (100 μg/ml) (XC‑A4122) (all Biosera) |
| Embryonal rhabdomyosarcoma | RD | CVCL_1649 | | ECACC | Post | DMEM High Glucose (LM‑D1112, Biosera) | 10% FBS (FB-1101); 2 mM L‑Glutamine (XC-T1715); 1× MEM Non-Essential Amino Acids (XC‑E1154); Penicillin (100 IU/ml)/Streptomycin (100 μg/ml) (XC‑A4122) (all Biosera) |
|  | NSTS-11 | In-house derived (under IGA MZCR NR/9125-4 project; ethics approval no. 23/2005) | | | Post | DMEM Low Glucose (LM‑D1100, Biosera) | 20% FBS (FB-1101); 2 mM L‑Glutamine (XC-T1715); Penicillin (100 IU/ml) /Streptomycin (100 μg/ml) (XC‑A4122) (all Biosera) |
|  | NSTS-46 |  |  |  | Pre |  |  |
| Human embryonic kidney | HEK293T | CVCL_0063 | ECACC | | - | DMEM Low Glucose (LM‑D1100, Biosera) | 10% FBS (FB-1101); 2 mM L‑Glutamine (XC-T1715); Penicillin (100 IU/ml) /Streptomycin (100 μg/ml) (XC‑A4122) (all Biosera) |

*Therapy status: ‘pre’ – cell line derived from therapy-naïve tumor; ‘post’ – cell line derived from the tumor sample after treatment. Providers: Biosera (Nuaille, France), Bovogen Biological (Keilor, Victoria, Australia); ECACC, The European Collection of Authenticated Cell Cultures, UK Health Security Agency); FBS, fetal bovine serum.
