# Supplementary material for: Depleting chemoresponsive mitochondrial fission mediator DRP1 does not mitigate sarcoma resistance
Source: Life Sci Alliance. 2024 Dec 6;8(2):e202402870. doi: 10.26508/lsa.202402870 (PMC11629689; doi:10.26508/lsa.202402870)
Supplement: Supplementary file 15 [file LSA-2024-02870_TableS2.docx]

**Supplementary Table 2. Drugs used in the study**

| **Drug** | **Manufacturer** | **Catalog number** | **Solvent** |
| --- | --- | --- | --- |
| ABT-737 | Abcam | ab141336 | DMSO |
| Bafilomycin-A1 | Sigma‒Aldrich | 19-148 | DMSO |
| Cisplatin | Sigma‒Aldrich | 232120 | PBS |
| Chloroquine | Tocris | 4109 | H_2_O |
| Doxorubicin | Tocris | 2252 | DMSO |
| Etoposide | Tocris | 1226 | DMSO |
| Phenformin hydrochloride | Sigma‒Aldrich | P7045 | H_2_O |
| Sunitinib | Cell Signaling | 12328S | DMSO |
| Trametinib | APEXBIO | GEN1590424 | DMSO |
| Topotecan | Accord Healthcare | 44/322/12-C | H_2_O |
| Vincristine | Tocris | 1257 | DMSO |

Providers: Abcam (Cambridge, UK), Accord Healthcare (London, UK), APEXBIO (Houston, TX, USA), CST – Cell Signaling Technology (Danvers, MA, USA), EMD Millipore (Billerica, MA, USA), Sigma‒Aldrich (St. Louis, MO, USA), Serva (Heidelberg, Germany), and Tocris (Bristol, UK).
