# Supplementary material for: Depleting chemoresponsive mitochondrial fission mediator DRP1 does not mitigate sarcoma resistance
Source: Life Sci Alliance. 2024 Dec 6;8(2):e202402870. doi: 10.26508/lsa.202402870 (PMC11629689; doi:10.26508/lsa.202402870)
Supplement: Supplementary file 16 [file LSA-2024-02870_TableS3.docx]

**Supplementary Table 3. Antibodies used for immunodetection**

| **Primary antibody (antigen)** | **Manufacturer** | **Catalog number** | **Blocking agent** | **Method** | **Dilution** | **Sample denaturation** |
| --- | --- | --- | --- | --- | --- | --- |
| AMPKα1 | CST | 5832 | NFM | WB | 1:1 000 | 95°C 5 min |
| AMPKα2 | CST | 2757 | NFM | WB | 1:1 000 | 95°C 5 min |
| phospho-AMPKα (Thr172) | CST | 50081 | NFM | WB | 1:1 000 | 95°C 5 min |
| CD133 | CST | 64326 | NFM | WB | 1:1 000 | 65°C 10 min |
| DRP1 | CST | 8570 | NFM | WB | 1:1 000 | 95°C 5 min |
| phospho-DRP1 (Ser616) | CST | 4494 | NFM | WB | 1:1 000 | 95°C 5 min |
| FIS1 | ProteinTech | 10956-1-AP | NFM | WB | 1:2 000 | 95°C 5 min |
| GAPDH | SCBT | sc-365062 | NFM | WB | 1:10 000 | 95°C 5 min |
| KLF4 | CST | 4038 | NFM | WB | 1:1 000 | 95°C 5 min |
| LC3A/B | CST | 12741S | NFM | WB | 1:2 000 | 95°C 5 min |
| MFF | CST | 84580 | BSA | WB | 1:2 000 | 95°C 5 min |
| Mitofusin-1 | CST | 14739 | BSA | WB | 1:2 000 | 95°C 5 min |
| Mitofusin-2 | CST | 9482 | NFM | WB | 1:1 000 | 95°C 5 min |
| OCT4 | CST | 2750 | NFM | WB | 1:1 000 | 95°C 5 min |
| OMA1 | CST | 95473 | NFM | WB | 1:1 000 | 95°C 5 min |
| OPA1 | CST | 80471 | BSA | WB | 1:2 000 | 95°C 5 min |
| p42/44 MAPK (ERK1/2) | CST | 4695 | NFM | WB | 1:2 000 | 95°C 5 min |
| Phospho-p42/44 MAPK (ERK1/2) (Thr202/Tyr204) | ProteinTech | 28733-1-AP | NFM | WB | 1:1 000 | 95°C 5 min |
| TOMM20 | SCBT | sc-17764 | BSA | IF | 1:100 | - |
| TOMM20 | CST | 42406 | NFM | WB | 1:3 000 | 95°C 5 min |
| YME1L1 | ProteinTech | 11510-1-AP | NFM | WB | 1:1000 | 95°C 5 min |
| α-Tubulin | ProteinTech | 66031-1-Ig | NFM | WB | 1:10 000 | 95°C 5 min |
| β-Actin | Sigma | A5441 | NFM | WB | 1:10 000 | 95°C 5 min |
| **Secondary antibody** | **Manufacturer** | **Catalog number** | **Blocking agent** | **Method** | **Dilution** |  |
| Anti-mouse IgG, HRP-linked | CST | 7076 | NFM | WB | 1:5 000; 1:10 0000 | |
| Anti-rabbit IgG, HRP-linked | CST | 7074 | NFM | WB | 1:5 000; 1:10 0000 | |
| Anti-mouse IgG Alexa Fluor ® 488 | Invitrogen* | A21202 | BSA | IF | 1:200 |  |
| **Isotype control antibody** | **Manufacturer** | **Catalog number** | **Blocking agent** | **Method** | **Dilution** |  |
| IgG2a Mouse Isotype Control | Thermo Fisher | MA1-10418 | BSA | IF | 1:500 |  |

Providers: Abcam (Cambridge, UK), CST – Cell Signaling Technology, Inc. (Danvers, MA, USA), Invitrogen (Carlsbad, CA, USA), Proteintech Group (Rosemont, IL, USA), SCBT – Santa Cruz Biotechnology, Inc. (Dallas, TX, USA), Sigma‒Aldrich (St. Louis, MO, USA), and Thermo Fisher Scientific (Waltham, MA, USA). BSA, bovine serum albumin; IF, immunofluorescence staining; NFM, dry nonfat milk; WB, western blotting.
